# Using non-Gaussian diffusion models to distinguish benign from malignant head and neck lesions

**DOI:** 10.3389/fonc.2025.1581637

**Published:** 2025-05-29

**Authors:** Li Hua, Qiuyang Guo, Yifan Tang, Xueyi Ding, Jianyu Lin, Mengxiao Liu, Jun Liu, Qing Yang

**Affiliations:** ^1^ Department of Laboratory Medicine, Anhui Medical University Anqing Medical Center, Anqing Municipal Hospital, Anqing, China; ^2^ Department of Medical Imaging, Anhui Medical University Anqing Medical Center, Anqing Municipal Hospital, Anqing, China; ^3^ Magnetic Resonance (MR) Search & Marketing Department, Siemens Healthineers Co., Ltd, Shanghai, China; ^4^ Department of Radiology, The First Hospital of Lanzhou University, Lanzhou, Gansu, China

**Keywords:** head and neck lesions, fractional-order calculus, continuous-time random walk, diffusion-weighted imaging, magnetic resonance imaging

## Abstract

**Objective:**

This study aims to investigate the application value of fractional-order calculus (FROC) and continuous-time random-walk (CTRW) derived multiple parameters in distinguishing benign and malignant head and neck lesions and compare their performance with conventional diffusion-weighted imaging (DWI).

**Methods:**

A retrospective analysis was conducted on 70 pathologically confirmed cases, including 23 benign lesions (BL) and 47 malignant lesions (ML). ML was further classified into lymphoma subgroups (LS, 11 cases, 15 lesions) and malignant lesions subgroups excluding lymphoma (MLS, 36 cases). DWI scans with 12 b-values were performed before treatment, and seven diffusion parameters—ADC, D_FROC_, β_FROC_, μ_FROC_, D_CTRW_, α_CTRW_, and β_CTRW_—were extracted from conventional DWI, FROC, and CTRW diffusion models. Independent t-tests or U-tests were used to compare parameter differences among BL, ML, LS, and MLS. Diagnostic performance was evaluated using receiver operating characteristic (ROC) curves, with area under the curve (AUC) compared via DeLong analysis. Pearson correlation analysis was conducted to explore relationships between diffusion parameters and Ki-67 expression in the MLS group.

**Results:**

ADC, D_FROC_, μ_FROC_, D_CTRW_, and α_CTRW_ showed significant differences between all groups, α_CTRW_ demonstrated the highest diagnostic performance (AUC). Significant correlations were found between Ki-67 expression and D_FROC_ (r = -0.367, *p* = 0.028), D_CTRW_ (r = -0.376, *p* = 0.024), α_CTRW_ (r = -0.418, *p* = 0.011), and β_CTRW_ (r = 0.525, *p* = 0.001).

**Conclusion:**

Multiple diffusion parameters derived from FROC and CTRW models effectively differentiate between benign and malignant head and neck lesions, reflecting tumor heterogeneity. Among them, α_CTRW_ showed the best diagnostic performance, making it a promising non-invasive imaging biomarker for quantitative assessment and differential diagnosis of head and neck tumors, thereby improving diagnostic accuracy.

## Introduction

Head and neck tumors, both benign and malignant, represent a diverse group of pathological entities. Accurate preoperative differentiation is vital for guiding individualized treatment strategies (1). Imaging serves as a cornerstone in the assessment of head and neck tumors, with computed tomography (CT) and magnetic resonance imaging (MRI) being the most frequently employed non-invasive modalities. CT, owing to its high spatial resolution, provides excellent visualization of anatomical structures and is particularly advantageous for evaluating bone involvement. However, its relatively poor soft-tissue contrast limits its capacity to fully characterize tumor composition. Recent advancements, including hyperspectral imaging and computer-aided diagnosis, have enhanced soft-tissue differentiation by enabling material decomposition and virtual monoenergetic imaging, offering improved accuracy in the quantitative analysis of iodine concentration (2). Nevertheless, the use of ionizing radiation in CT poses concerns, especially for patients requiring serial follow-up, thereby limiting its routine clinical use. In contrast, MRI offers superior soft-tissue contrast and provides valuable information regarding tumor localization and invasion of adjacent structures. Despite these advantages, MRI interpretation remains highly dependent on radiologist expertise and lacks standardized quantitative diagnostic criteria (3).

The conventional apparent diffusion coefficient (ADC), which quantitatively evaluates the microstructure of tumor tissues (4), has been widely used to differentiate between benign and malignant head and neck lesions (5, 6). This model assumes that the diffusion of water molecules occurs in a homogeneous environment. However, in tumor tissues with high structural heterogeneity, where simple ADC values are unable to adequately describe the tissue heterogeneity (7). Given the heterogeneity of biological tissues, especially tumors that exhibit a high degree of structural heterogeneity and complexity, it is widely accepted that water molecule diffusion in such tissues does not follow a Gaussian distribution (8). While some studies have reported significant differences between benign and malignant groups (9), others show considerable overlap between different types of tumors (10).

To address these limitations, non-Gaussian diffusion models have been developed to better capture tissue microstructure and heterogeneity. The fractional order calculus (FROC) model, based on the Bloch-Torrey equation (11), generates three parameters that describe the complex diffusion process in heterogeneous tumor tissues: diffusion coefficient (D_FROC_, μm²/ms), spatial fractional order derivative (β_FROC_), and spatial parameter (μ_FROC_, μm). These parameters provide insights into diffusion dynamics (D_FROC_), structural complexity (β_FROC_), and the diffusion environment (μ_FROC_). Similarly, the continuous-time random walk (CTRW) is another interesting model (12) that provides two parameters related to intra-voxel tissue structural heterogeneity: temporal diffusion heterogeneity (a_CTRW_) and spatial diffusion heterogeneity (β_CTRW_), thus providing a method for studying changes in tumor structure. Additionally, the derived parameter, D_CTRW_ (μm²/ms), is similar to ADC, measures abnormal diffusion processes, and is sensitive to tissue cellularity.

Previous studies have demonstrated the value of the FROC and CTRW models in differentiating lesions of the nervous system, breast, prostate, and bladder (11–22). However, no study to date has delved into the application of high spatial resolution FROC and CTRW diffusion in the differentiation of head and neck tumors. Therefore, the purpose of this study was to evaluate the value of these two non-Gaussian models for the differentiation of head and neck tumors and analyze the relationship between each diffusion parameter and Ki-67 expression in squamous cell carcinoma.

## Materials and methods

### Patients

This retrospective study, approved by our hospital’s medical ethics committee, was designed with a waived consent process due to its retrospective nature. From January 2022 to July 2024, 70 patients with pathologically confirmed head and neck tumors were retrospectively analyzed. The inclusion criteria were as follows: (1) MRI scans performed before or within three days of biopsy without receiving relevant treatment, and without a history of head and neck cancer; (2) All patients were diagnosed by tissue biopsy or postoperative pathology and tested for Ki-67 marker expression; (3) scans included multi-b-value diffusion sequences; (4) The minimum short diameter of the lesion is > 1 cm. The exclusion criteria were defined as follows: (1) inflammatory lesions or lipomas; (2) underwent radiotherapy or chemotherapy before MRI scan; (3) severe image artifacts or small lesions caused difficulties in delineating the region of interest (ROI); (4) Excessive areas of necrosis, hemorrhage, or cystic degeneration are present within the lesion.

Immunohistochemical assessment of Ki-67 expression was performed as follows. Tissue specimens were fixed in 10% neutral buffered formalin for 18–36 hours, followed by dehydration and paraffin embedding. Two consecutive 3-μm-thick sections were cut from each paraffin block. One section was subjected to hematoxylin and eosin staining for histopathological classification and tumor grading. The other section underwent immunohistochemical staining for Ki-67. After dewaxing and rehydration, antigen retrieval was performed using ethylenediaminetetraacetic acid buffer. The sections were then incubated with the primary antibody at room temperature for 60 minutes, followed by incubation with the secondary antibody for 20 minutes. Diaminobenzidine was used for chromogenic detection, and the sections were subsequently counterstained with hematoxylin, blued, dehydrated through graded alcohols, cleared in xylene, and mounted with a coverslip. For quantification, five randomly selected high-power fields (×400) were examined. Tumor cells with brownish-yellow nuclear staining were considered Ki-67 positive. In each field, 200 tumor cells were counted, and the proportion of positively stained cells was calculated. The average of the five fields was used to determine the Ki-67 labeling index, expressed as: Ki-67 labeling index (%) = (number of positively stained cells/total number of tumor cells) × 100%.

### MR acquisitions

#### MR imaging protocol

All MR examinations were performed using a 3.0T whole-body scanner (MAGNETOM Vida, Siemens Healthcare, Erlangen, Germany) equipped with a 20-channel head and neck phased-array coil.

The routine imaging protocol included the following sequences:

1. Coronal T2-weighted imaging with fat saturation (T2WI-FS): repetition time (TR) = 4110 ms; echo time (TE) = 94 ms; field of view (FOV) = 28cm × 28cm; slice thickness = 4 mm; inter-slice gap = 1 mm; matrix size = 320 × 224; number of excitations (NEX) = 2; bandwidth = 401 Hz; echo spacing = 9.42 ms.
2. Axial T1-weighted imaging (T1WI): TR = 468 ms; TE = 6.5 ms; FOV = 22cm × 22cm; slice thickness = 4 mm; inter-slice gap = 1 mm; matrix = 320 × 224; NEX = 3; bandwidth = 391 Hz; echo spacing = 6.53 ms.
3. Axial T2-weighted imaging with fat saturation (T2WI-FS): TR = 4170 ms; TE = 96 ms; FOV = 22cm × 22cm; slice thickness = 4 mm; inter-slice gap = 1 mm; matrix = 320 × 224; NEX = 2; bandwidth = 381 Hz; echo spacing = 9.56 ms.
4. Diffusion-weighted imaging (DWI) was performed using a readout-segmented echo-planar imaging sequence (RESOLVE) with 12 b-values (0, 10, 20, 50, 100, 200, 400, 800, 1000, 1500, 2000, and 3000 s/mm²), with a single excitation for each b-value. Imaging parameters were as follows: TR = 5200 ms; TE = 70 ms; slice thickness = 4 mm; inter-slice gap = 20% of slice thickness; FOV = 22cm × 22cm; partial Fourier = 6/8; number of slices = 22; diffusion mode = 3-scan trace; readout segments = 5; bandwidth = 930 Hz; echo spacing = 0.36 ms; acquisition time = 11 min 49 s.

Finally, contrast-enhanced axial, coronal, and sagittal T1-weighted images were obtained after intravenous injection of 0.1 mmol/kg of gadolinium-DTPA (Gd-DTPA) via the median cubital vein at a rate of 2 mL/s, followed by a 20 mL saline flush.

### Imaging analysis

The conventional DWI, FROC, and CTRW diffusion images were processed using the Body DiffusionLab (BoDilab, Chengdu ZhongYing Medical Technology Co., Ltd., Chengdu,CN) software in the MR workstation.

For conventional DWI, the quantitative parameter ADC was generated by fitting a mono-exponential model (**Equation 1**):


(1) $$\frac{S\left(b\right)}{S\left(0\right)}=exp\left(-b\cdot ADC\right)$$

The FROC model is given by **Equation 2**:


(2) $$\frac{S\left(b\right)}{S\left(0\right)}=exp\left[-D\mu^{2\left(\beta -1\right)}{\left(\gamma G_{d\delta }\right)}^{2\beta }\left(\text{Δ}-\frac{2\beta -1}{2\beta +1}\delta \right)\right]$$

where S0 is the signal intensity without diffusion weighting, G_d_ is the diffusion gradient amplitude, δ is the diffusion gradient pulse width and Δ is the gradient interval. The β (dimensionless; 0 < β ≤ 1) parameter is the intra-voxel diffusion heterogeneity parameter, and μ (unit: μm) is a spatial constant to maintain the unit of diffusion coefficient D (unit: μm^2^/ms). The multi-b-value diffusion images were fitted pixelwise to the FROC diffusion model using the Levenberg-Marquardt nonlinear fitting algorithm, in which D (reflecting the intrinsic diffusion coefficient) was estimated using a mono-exponential model and data were acquired at lower b-values (≤ 1000 s/mm^2^). After determining the D_FROC_, β and μ were obtained by performing pixel-wise nonlinear fitting using all b-values.

The CTRW model was fitted using **Equation 3**:


(3) $$\frac{S\left(b\right)}{S\left(0\right)}=E_{\alpha }\left[{\left(-bD\right)}^{\beta }\right]$$

where D is the anomalous diffusion coefficient, α and β are parameters related to temporal and spatial diffusion heterogeneity, respectively, and E_α_ is the Mittag-Leffler function. D_CTRW_ was first estimated through the nonlinear fitting of diffusion images with b-values less than 1000 ms/mm^2^, then α and β were determined simultaneously from all diffusion-weighted images (b-values = 0–3000 s/mm^2^).

Two observers with 5 and 15 years of experience in diagnosing head and neck tumor diagnosis independently evaluated all parameters in a double-blind manner, transcending potential bias. Using dynamic contrast-enhanced or T2WI images as reference, the DWI images with the best lesion signal intensity contrast were selected to manually delineate the ROIs, while avoiding necrotic areas, air, major blood vessels, and adjacent anatomical structures. The software automatically transferred the delineated ROIs to the parameter maps and obtained the calculation results. The mean values of the two observers’ measurements were taken as final values for all parameters and the interclass correlation coefficients between the two observers were calculated. The same observer repeated the measurements after one week to calculate the intraclass correlation coefficients.

### Statistical analysis

Quantitative parameters were tested for normality and parameters were expressed as mean ± standard deviation ( $\bar{\text{x}}$ ± S). SPSS v.23.0 (IBM Corp, Armonk, NY), GraphPad Prism v.8.0 (GraphPad Software, San Diego, CA), and MedCalv.15.11.4 (MedCalc Software, Mariakerke, Belgium) was used for data analysis. Differences in the parameters between each group were analyzed by independent samples t-tests or U-tests. Receiver operating characteristic (ROC) curves were employed to analyze the diagnostic performance of each parameter in predicting benign and malignant lesions. The DeLong test was performed to compare the area under the curve (AUC) between the two groups. The relationship between each diffusion parameter and Ki-67 in the MLS group was analyzed by Pearson’s correlation analysis. Inter-observer agreement was defined by the intraclass and interclass correlation coefficients within the 95% confidence interval. Two-tailed P ≤ 0.05 indicates the difference was statistically significant.

## Results

**Figure 1:**
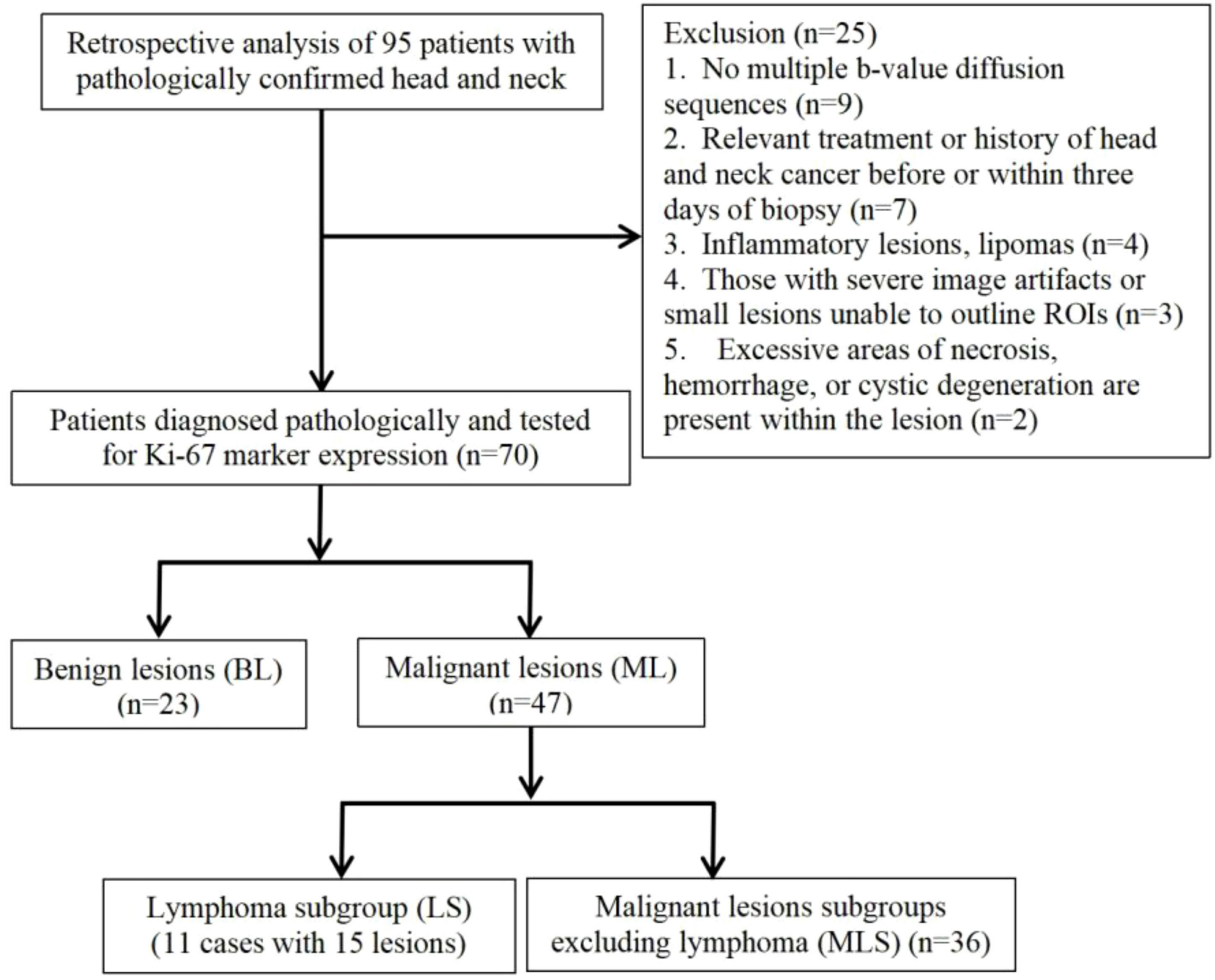
Flowchart of enrolled cases.

**Figure 2:**
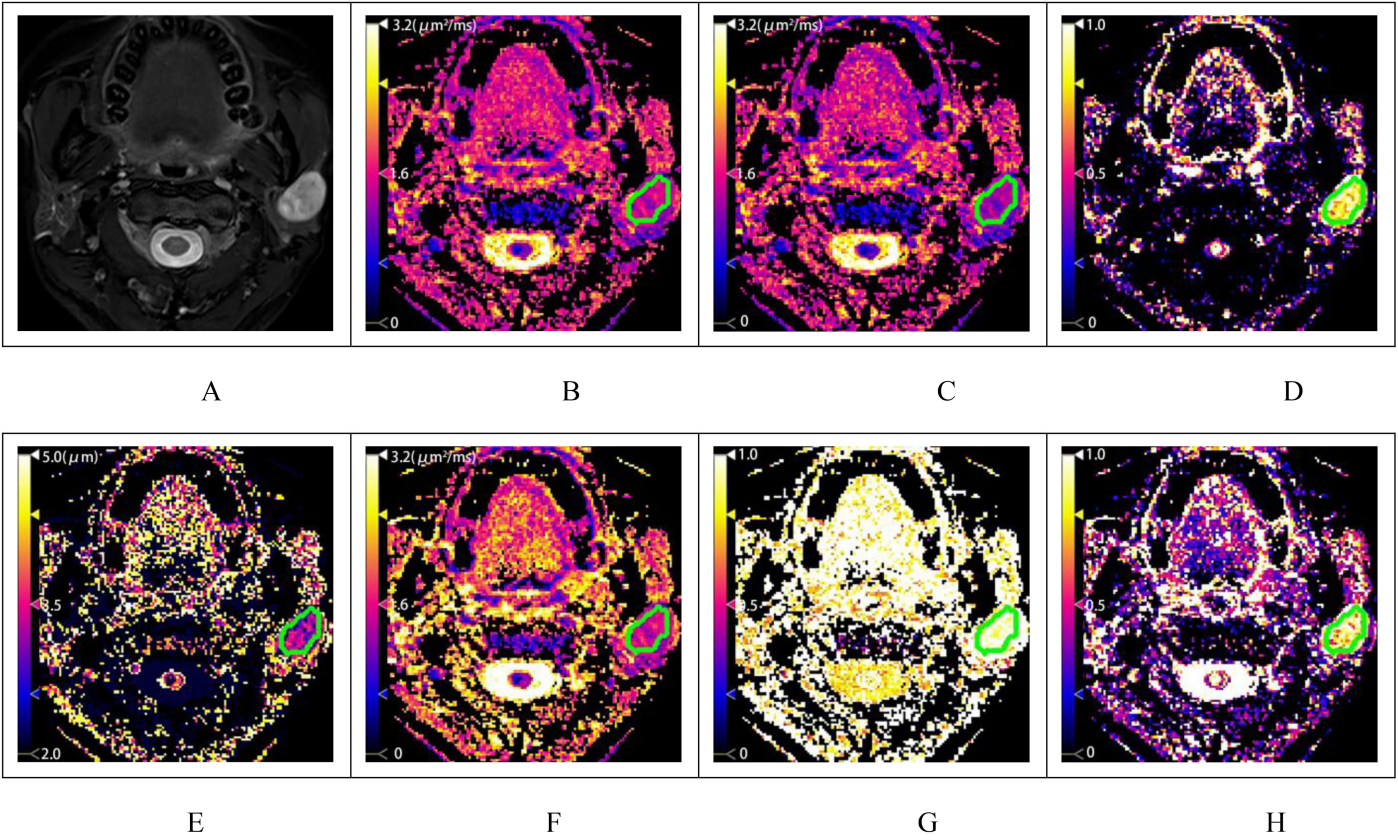
A patient with pleomorphic adenoma of the left parotid gland. **(A)** shows the T2-weighted fat-saturation image, and **(B–H)** display the diffusion-weighted image (DWI), fractional anisotropy (FROC), and color maps of the CTRW model parameters, respectively. The measured values are as follows: ADC=1.510μm^2^/ms (2B), D_FROC_=1.203μm^2^/ms **(C)**, β_FROC_=0.888 **(D)**, μ_FROC_=3.021μm **(E)**, D_CTRW_=1.412μm^2^/ms **(F)**, α_CTRW_=0.971 **(G)** and β_CTRW_=0.891 **(H)**.

**Figure 3:**
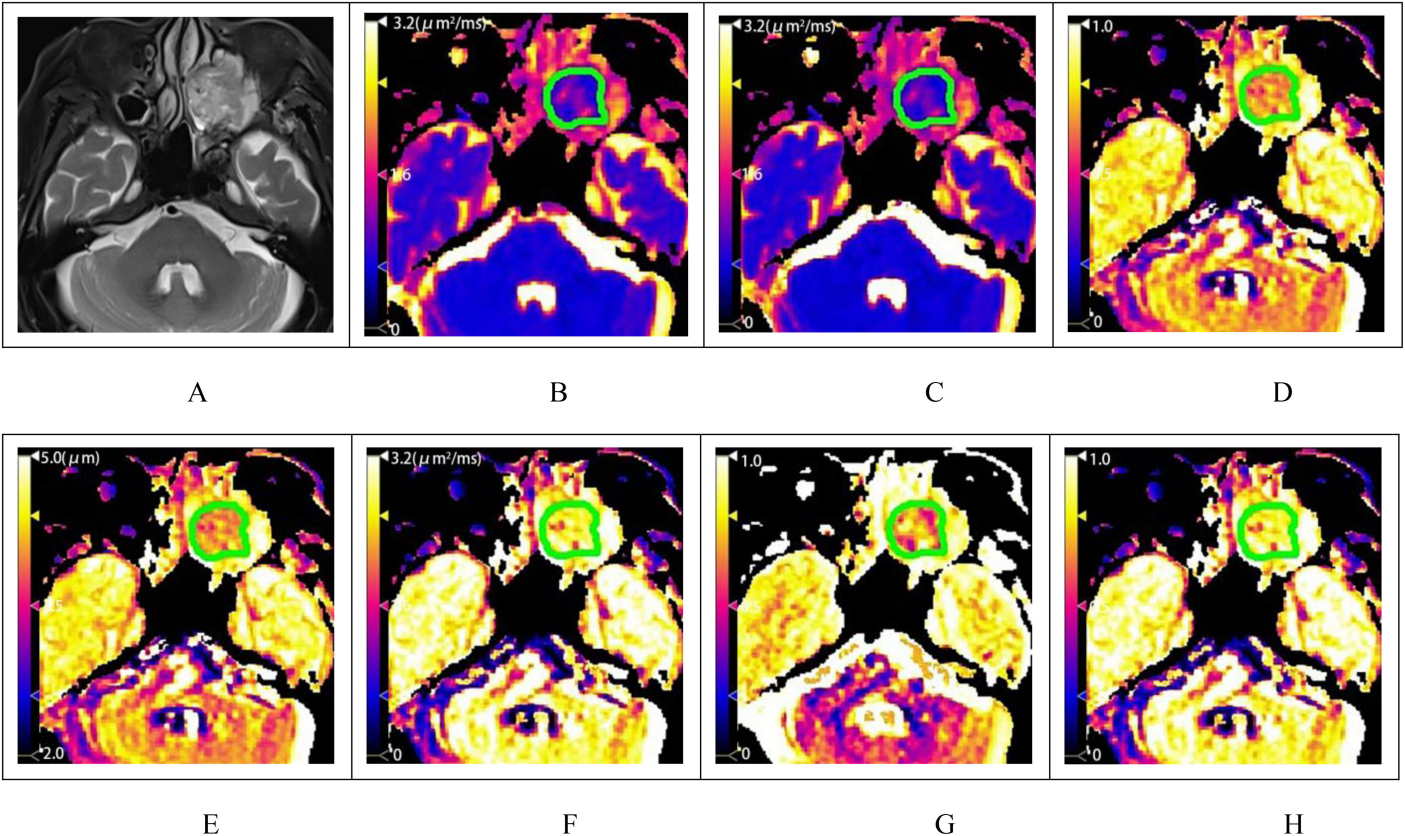
A patient with squamous cell carcinoma of the left maxillary sinus. **(A)** shows the T2-weighted fat-saturation image, and **(B–H)** display the pseudo-color maps of DWI, FROC, and CTRW model parameters, respectively. The measured values are as follows: ADC=0.963μm^2^/ms **(B)**, D_FROC_=0.903μm^2^/ms **(C)**, β_FROC_=0.780 **(D)**, μ_FROC_=3.505μm **(E)**, D_CTRW_=1.219μm^2^/ms **(F)**, α_CTRW_=0.795 **(G)** and β_CTRW_=0.875 (3H).

1. Lesion Grouping: Among the 70 patients with head and neck tumors finally enrolled in the study, 23 had benign lesions (BL), including 11 cases of pleomorphic adenomas, 2 cases of hemangiomas, 2 cases of inverted papillomas, 2 cases of nasal polyps, 3 cases of lymphoid hyperplasia, 1 case of basal cell adenoma and 2 cases of schwannomas; 47 patients had malignant lesions (ML), ML was further divided into the lymphoma subgroup (LS) (11 cases of lymphomas with 15 lesions) and malignant lesion subgroup excluding lymphoma (MLS) (36 cases of squamous cell carcinoma). The patient enrollment process is shown in **Figure 1**; patient clinical information is presented in **Table 1**; examples of multi-parameter imaging and measurements of the lesions are shown in **Figure 2** and **3**.

**Table 1:** Patient and pathological characteristics of benign and malignant lesions.

Characteristics	Benign Group (23)	Malignant Group (47)	*p*
Demographics			
Male/female	12/11	27/20	0.307
Age (y)	56.2 ± 19.2	60.9 ± 14.0	0.547
Pathological results and location	Pleomorphic Adenoma	Squamous cell carcinoma (36)	
	Parotid Gland (11)	Nasopharynx (20)	
	Lymphoid Hyperplasia	Paranasal sinus (8)	
	Nasopharynx (3)	Tongue (4)	
	Hemangioma	Palate (4)	
	Tongue (1)	Lymphoma (11)	
	Palate (1)	Paranasal sinus (4)	
	Basal Cell Adenoma	Nasopharynx (3)	
	Parotid Gland (1)	Neck (3)	
	Inverted Papilloma		
	Sinus (2)		
	Nasal Polyp		
	Sinus (2)		
	Schwannoma		
	Parapharyngeal Space (2)		

2. Inter-observer agreement of parameters: The interclass correlation coefficients (95% CI) of the quantitative parameters D_FROC,_ β_FROC_, μ_FROC_, D_CTRW_, α_CTRW,_ and β_CTRW_ for inter-observer reproducibility ranged from 0.801 to 0.950, while the intraclass correlation coefficients (95% CI) for intra-observer reproducibility ranged from 0.885 to 0.959. These results indicate that the parameters had good inter- and intra-observer reproducibility and consistency (**Table 2**).

**Table 2:** Reproducibility of DWI parameters.

Parameters	Intra (95%CI)	Inter (95%CI)
ADC (μm^2^/ms)	0.885 (0.817-0.928)	0.913 (0.862-0.945)
D_FROC_ (μm^2^/ms)	0.958 (0.933-0.973)	0.939 (0.902-0.961)
β_FROC_	0.912 (0.860-0.945)	0.801 (0.685-0.875)
μ_FROC_ (μm)	0.923 (0.875-0.953)	0.848 (0.759-0.904)
D_CTRW_ (μm^2^/ms)	0.910 (0.857-0.943)	0.928 (0.886-0.955)
α_CTRW_	0.902 (0.844-0.938)	0.906 (0.850-0.941)
β_CTRW_	0.959 (0.934-0.975)	0.950 (0.918-0.969)

**Figure 4:**
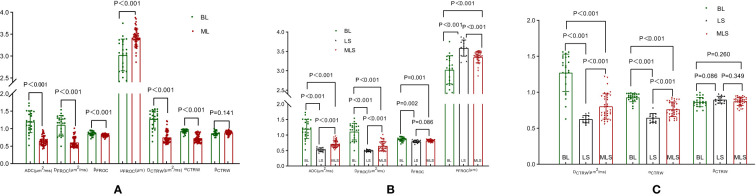
Box plots of DWI, FROC, and CTRW diffusion model parameters between benign and malignant lesions **(A)**; box plots of differences in DWI, FROC diffusion model parameters between BL, LS, and MLS **(B)**; box plots of differences in CTRW diffusion model parameters between BL, LS, and MLS **(C)**. DWI, diffusion-weighted imaging; ADC, apparent diffusion coefficient; FROC, fractional-order calculus; CTRW, continuous-time random walk; BL, benign lesion; ML, malignant lesion; LS, lymphoma subgroup; MLS, malignant lesions excluding lymphoma subgroup.

3. Parameter Differences between Groups: Among the BL, ML, LS, and MLS groups, all differences were statistically significant for ADC, D_FROC_, μ_FROC_, D_CTRW,_ and α_CTRW_. The differences in β_FROC_ were statistically significant for BL vs. ML, BL vs. LS, and BL vs. MLS; whereas the differences in β_CTRW_ were not statistically significant between the groups (**Tables 3**, **4**, **Figure 4**).

**Table 3:** Differences in parameters between benign and malignant head and neck lesions.

	ADC (μm^2^/ms)	D_FROC_ (μm^2^/ms)	β_FROC_	μ_FROC_ (μm)	D_CTRW_ (μm^2^/ms)	α_CTRW_	β_CTRW_
BL	1.185 ± 0.309	1.105 ± 0.275	0.860 ± 0.067	3.026 ± 0.369	1.324(1.085-1.489)	0.940(0.897-0.975)	0.856(0.817-0.917)
ML	0.658 ± 0.129	0.550(0.487-0.721)	0.809 ± 0.038	3.422 ± 0.195	0.703(0.623-0.898)	0.702(0.636-0.833)	0.889(0.852-0.925)
T/Z	-5.903	-5.819	-3.523	-4.653	-5.819	-6.023	-1.473
*p*	<0.001	<0.001	<0.001	<0.001	<0.001	<0.001	0.141

BL, Benign lesion; ML, malignant lesions.

**Table 4:** Difference in each parameter between benign and malignant subgroups.

Parameters	BL	LS	MLS	*p*		
				BL-LS	BL-MLS	LS-MLS
ADC (μm^2^/ms)	1.185 ± 0.309	0.530 ± 0.057	0.711 ± 0.112	<0.001	<0.001	<0.001
D_FROC_ (μm^2^/ms)	1.105 ± 0.275	0.497 ± 0.031	0.602(0.533-0.767)	<0.001	<0.001	<0.001
β_FROC_	0.860 ± 0.067	0.795 ± 0.043	0.815 ± 0.034	0.002	0.001	0.086
μ_FROC_ (μm)	3.026 ± 0.369	3.588 ± 0.202	3.384(3.301-3.457)	<0.001	<0.001	<0.001
D_CTRW_ (μm^2^/ms)	1.324(1.085-1.489)	0.623 ± 0.053	0.745(0.674-0.945)	<0.001	<0.001	<0.001
α_CTRW_	0.940(0.897-0.975)	0.641 ± 0.061	0.747(0.691-0.855)	<0.001	<0.001	<0.001
β_CTRW_	0.864 ± 0.067	0.899 ± 0.045	0.886(0.846-0.919)	0.086	0.349	0.260

BL, Benign lesion; LS, Lymphoma Subgroup; MLS, malignant lesion subgroup excluding lymphoma; ADC, apparent diffusion coefficient; FROC, fractional order calculus; CTRW, continuous-time random walk.

**Figure 5:**
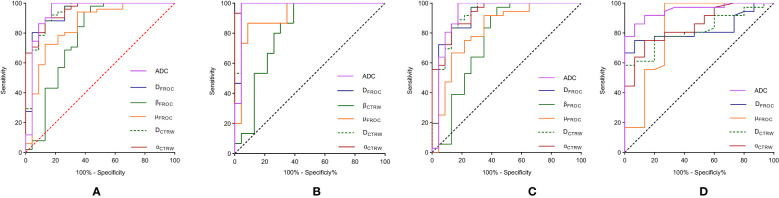
ROC curves of DWI, FROC, and CTRW diffusion model parameters in the diagnosis of benign and malignant **(A)**, BL vs. LS **(B)**, BL vs. MLS **(C)** and LS vs. MLS **(D)**. DWI, diffusion-weighted imaging; ADC, apparent diffusion coefficient; FROC, fractional-order calculus; CTRW, continuous-time random walk; BL, benign lesions; ML, malignant lesions; LS, lymphoma subgroup; MLS, malignant lesions excluding lymphoma subgroup.

4. Diagnostic performance and comparisons of parameters between BL vs. ML, LS and MLS, and LS vs. MLS are summarized in **Table 5**. ROC curve analysis showed that α_CTRW_ had the best performance in differentiating between BL vs. ML (AUC = 0.947), while the diagnostic performance of ADC, D_FROC_, D_CTRW_ and α_CTRW_ differed significantly from that of β_FROC_ (*p* = 0.024, 0.018, 0.031, and 0.006, respectively) (**Figure 5A**).

For BL vs. LS, α_CTRW_ again exhibited the best diagnostic efficacy (AUC = 0.997), with ADC, D_FROC_, D_CTRW_, and α_CTRW_ outperforming β_FROC_ (*p* = 0.024, 0.018, 0.018, and 0.008, respectively; **Figure 5**).
In differentiating BL from MLS, α_CTRW_ achieved the best performance (AUC = 0.929), and its diagnostic ability, along with that of ADC and D_FROC_, was significantly higher than that of β_FROC_ (*p* = 0.031, 0.025, and 0.009, respectively; **Figure 5**).

**Table 5:** ROC results of each parameter in differential diagnosis of head and neck lesions.

Parameters	AUC	Thresholds	YI	Sensitivity (%)	Specificity (%)	AUC	Thresholds	YI	Sensitivity (%)	Specificity (%)
	BL vs. ML					BL vs. LS				
ADC (μm^2^/ms)	0.940	0.967	0.826	100.00	82.61	0.971	0.670	0.955	100.00	95.45
D_FROC_ (μm^2^/ms)	0.931	0.745	0.759	80.39	95.45	0.977	0.654	0.955	100.00	95.45
β_FROC_	0.761	0.858	0.532	94.12	59.09	0.795	0.858	0.591	100.00	59.09
μ_FROC_ (μm)	0.845	3.369	0.589	72.55	86.36	0.912	3.391	0.766	85.71	90.91
D_CTRW_ (μm^2^/ms)	0.931	0.963	0.746	88.24	86.36	0.981	0.758	0.955	100.00	95.45
α_CTRW_	0.947	0.885	0.766	90.2	86.36	0.997	0.776	0.955	100.00	95.45
	BL vs. MLS					LS vs. MLS				
ADC (μm^2^/ms)	0.928	0.967	0.826	100.00	82.61	0.953	0.608	0.794	93.33	86.11
D_FROC_ (μm^2^/ms)	0.917	0.907	0.711	97.22	73.91	0.825	0.537	0.683	93.33	75.00
β_FROC_	0.756	0.865	0.537	97.22	56.52	/				
μ_FROC_ (μm)	0.821	3.164	0.569	91.67	65.22	0.831	3.530	0.733	73.33	100.00
D_CTRW_ (μm^2^/ms)	0.915	0.989	0.715	88.89	82.61	0.818	0.717	0.583	100.00	58.33
α_CTRW_	0.929	0.885	0.731	86.11	86.96	0.85	0.693	0.617	86.67	75.00

BL, Benign lesion; LS, Lymphoma Subgroup; MLS, malignant lesion subgroup excluding lymphoma; ADC, apparent diffusion coefficient; FROC, fractional order calculus; CTRW, continuous-time random walk.

For LS vs. MLS, ADC demonstrated the highest diagnostic performance (AUC = 0.953). Its performance was significantly superior to D_FROC_, D_CTRW_, and α_CTRW_ (*p* = 0.008, 0.011, and 0.015, respectively; **Figure 5**).

**Table 5** provides a detailed summary of these diagnostic comparisons.

**Figure 6:**
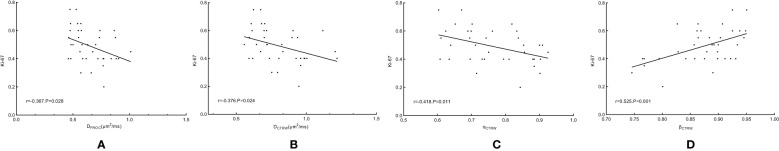
Scatterplot of correlation coefficients between D_FROC_ **(A)**, D_CTRW_ **(B)**, α_CTRW_ **(C)** and β_CTRW_ **(D)** and Ki-67. FROC, fractional-order calculus; CTRW, continuous-time random walk.

5. Correlation with Ki-67 Expression: The correlation between each diffusion parameter and Ki-67 expression level in 36 cases with head and neck squamous cell carcinoma was further analyzed. The results showed that the correlation coefficients r of Ki-67 with D_FROC_, D_CTRW_, α_CTRW_ and β_CTRW_ were -0.367 (*p* = 0.028), -0.376 (*p* = 0.024), -0.418 (*p* = 0.011) and 0.525(*p* = 0.001), respectively. While the correlation coefficients r of Ki-67 with ADC, β_FROC_ and μ_FROC_ were -0.276 (*p* = 0.103), 0.252 (*p* = 0.139), and 0.144 (*p* = 0.402), respectively (**Figure 6**).

## Discussion

In this study, we examined the clinical value of FROC, CTRW, and conventional DWI diffusion parameters for benign and malignant lesions of the head and neck. Our findings revealed that: Multi-parameters derived from the FROC and CTRW diffusion models can be used to differentiate between benign and malignant lesions of the head and neck and provide indicators that reflect tissue heterogeneity. Moreover, some of the diffusion parameters correlated with the expression level of Ki-67 in the pathological findings.

DWI is a powerful tool for exploring biological microstructures, with the ability to elucidate the cell number, extracellular matrix, vascular distribution, and microstructure of tumor tissues. ADC is derived from a mono-exponential diffusion model, which assumes that the diffusion-driven displacement of water molecules follows a Gaussian distribution. Our study found that ADC showed good diagnostic performance in the differential diagnosis of benign and malignant lesions of the head and neck, which is consistent with previous studies (5, 6). However, it oversimplifies the anomalous diffusion process in complex biological tissues and fails to recognize the heterogeneity of intra-voxel structures. As the complexity of tissue structure increases, this assumption becomes increasingly invalid (13). Unlike the mono-exponential diffusion model, the FROC model recognizes this heterogeneity of diffusion through its three parameters, namely, D_FROC_, β_FROC,_ and μ_FROC._ D_FROC_ is obtained by fitting the FROC model with multiple b-values less than 1000, which better reflects the true diffusion process in tissues. In this study, D_FROC_ showed good diagnostic performance for BL vs. ML, LS and MLS, and LS vs. MLS, which is similar to the findings in breast cancer (14). Previous studies have shown that β_FROC_ values are negatively correlated with increased intra-voxel heterogeneity (11, 15), which is due to the higher tissue heterogeneity of ML, leading to lower β_FROC_ values. Our findings indicated that BL had a greater β_FROC_ value than ML, and differences in β_FROC_ values were also observed between BL vs. LS and BL vs. MLS. μ_FROC_ is regarded as a measure of the mean free path of diffusion, with which it is negatively correlated (16). Malignant tumors present higher μ_FROC_ values as the abnormal proliferation of tumor cells can restrict the free diffusion of water molecules. Studies have shown that malignant breast lesions and high-grade bladder tumors exhibit higher μ_FROC_ values (14, 16). In this study, the μ_FROC_ value of BL was lower, which is in agreement with the results of previous studies. Lymphomas are tumors characterized by high cell density and low microvascularity, with histopathological features that include cellular hyperplasia, larger and irregular nuclei, limited extracellular space, and cellular compartments composed of lymphoma cells and fine fibers. As such, they exhibit more pronounced diffusion restriction and shorter diffusion mean free paths, which in turn manifests as lower D_FROC_ and higher μ_FROC_. Hence, lymphomas are often grouped separately for further evaluation (23). This was verified by our findings and is also consistent with previous studies demonstrating the lower ADC values of lymphomas (24, 25).

The CTRW model is another advanced diffusion MRI technique that describes non-Gaussian behavior (17–19). The model introduces two new parameters, α_CTRW_ and β_CTRW_, which denote temporal and spatial diffusion heterogeneity, respectively. In a homogeneous medium, α_CTRW_ and β_CTRW_ are close to 1, whereas the presence of tissue heterogeneity causes them to decrease. Smaller α_CTRW_ values indicate that the water molecules are diffusing through a more temporally inhomogeneous environment (i.e., the time taken for water molecules to move is variable), while larger β_CTRW_ values indicate a more spatially homogeneous environment (i.e., the water molecules diffuse in more uniform step sizes for each movement) (18, 19). In this study, BL was shown to have relatively high α_CTRW_, with differences in α_CTRW_ values found among the BL, LS, and MLS groups. Furthermore, α_CTRW_ had the highest diagnostic performance for BL vs. ML, LS, and MLS, and for LS vs. MLS, which was similar to high-grade gliomas with relatively high α_CTRW_ values (15). Theoretically, β_CTRW_ values should be negatively correlated with tissue heterogeneity, but the β_CTRW_ values in this study did not show significant differences between BL vs. ML, LS and MLS, and LS vs. MLS. This, on the one hand, may be due to the limited sample size, which led to the failure to achieve statistically significant differences. On the other hand, it may be because the different diffusion parameters reflect different aspects of tissue heterogeneity. For example, in studies involving vessels encapsulating tumor clusters in hepatocellular carcinoma and different subtypes of breast lesions (20, 21), only α_CTRW_ showed differences, whereas the β_CTRW_ did not. In contrast, in another study that assessed whether the muscle of bladder cancer was invaded or not (22), only β_CTRW_ showed a difference, and α_CTRW_ values did not differ between the two. This suggests that α_CTRW_ and β_CTRW_ can characterize changes in different properties of water molecule diffusion within the lesion. Thus, we can speculate that the diffusion heterogeneity of water molecules in the head and neck lesions included in this study may manifest more significantly as temporal differences. However, the specific mechanism involved remains unclear and awaits further investigation in future studies. The CTRW model also has an important derived parameter, D_CTRW_, which is similar to ADC (17, 21) and serves as a measure of tissue cell density. In this study, BL showed higher D_CTRW_, which was due to the higher cell density of ML leading to lower D_CTRW_ values.

Single-shot echo-planar imaging is commonly used for signal acquisition in DWI scans. Despite its fast-scanning speed, it is prone to geometric distortion and image blurring. It is also restricted by the high degree of heterogeneity present in the components of the head and neck region, is very sensitive to magnetic susceptibility artifacts at tissue interfaces, and is limited by its maximum resolution (26). The RESOLVE diffusion technique can shorten the echo gap by acquiring signals in the gradient direction in segments, thereby reducing the geometric distortion and T2* blurring of DWI to improve the anatomical accuracy of images. This technique has gradually been adopted for the evaluation of head and neck tumors (27, 28). Therefore, in this study, image acquisition was performed using RESOLVE to reduce distortions and artifacts in DWI images (28), and hence obtain more stable data. The results showed that the ICC of the diffusion parameters obtained ranged from 0.801 to 0.959, which showed very good agreement. Further comparative analysis of ROC curves showed that α_CTRW_ had the best diagnostic performance for BL vs. ML, LS, and MLS; α_CTRW_ did not differ from ADC, D_FROC_, μ_FROC_, and D_CTRW_, but differed from β_FROC_. ADC showed better diagnostic performance for LS vs. MLS. These findings suggest that the FROC and CTRW models can provide more diffusion parameters for the assessment of head and neck tumors.

Ki-67 is a nuclear antigen expressed by proliferating cells and is confined to the G1 to M phases of the cell cycle. It is involved in cell mitosis, and its positive expression is closely related to cell proliferation activity. Thus, it is widely used to assess the proliferation activity of tumor cells and can serve as an important indicator for determining the local recurrence, lymph node metastasis, distant metastasis, and poor prognosis of malignant tumors (29, 30). In general, ADC values are negatively correlated with Ki-67 (31, 32), but results may vary across different lesions. For example, a study of nasopharyngeal carcinoma confirmed that there was no correlation between ADC and Ki-67 (33), which is similar to the absence of correlation between ADC and Ki-67 found in this study. Further analysis of the relationship of FROC and CTRW parameters with Ki-67 parameters revealed that Ki-67 showed a low to moderate correlation with D_FROC_, D_CTRW_, α_CTRW,_ and β_CTRW_, with correlation coefficients r of -0.367, -0.376, -0.418, and 0.525, respectively; β_FROC_ and μ_FROC_ were not correlated with Ki-67. This was due to the increased number of intratumoral cells with high Ki-67 expression and decreased extracellular space, which resulted in lower D_FROC_ and D_CTRW_ values. The α_CTRW_ and β_CTRW_ parameters, which are indicators of tissue heterogeneity, showed a higher correlation with Ki-67, which suggests that non-Gaussian diffusion models can provide more reference information for the clinical treatment of tumors. Although the differences in AUC values among the diffusion-derived parameters were relatively small, α_CTRW_ consistently exhibited superior diagnostic performance across all classification tasks. This consistent superiority suggests that α_CTRW_ may serve as a robust imaging biomarker. Its moderate correlation with Ki-67 further supports its potential to reflect tumor proliferative activity, highlighting its clinical value in non-invasive tumor characterization. Interestingly, a previous study investigating cervical cancer demonstrated that β_CTRW_ was an independent predictor of the Ki-67 proliferation index, significantly improving the predictive accuracy of the combined model (34). This finding implies that β_CTRW_ may be related to tumor cell proliferation or microstructural remodeling. Taken together, these results suggest that non-Gaussian diffusion models could offer additional biological insights into tumor characterization and potentially support clinical decision-making. Nevertheless, the current evidence remains limited, and further well-designed studies are warranted to validate these findings and clarify the underlying mechanisms. However, limited research has been conducted on the association between the two, and further investigations are needed.

There are several limitations to this study. First, this is a single-center study with a small sample size. The value of non-Gaussian FROC and CTRW models for benign and malignant lesions of the head and neck region requires further exploration through multi-center studies with larger sample sizes. Second, no direct correlation analysis was performed between the MR images and the tissue sections. Although there were significant differences in several diffusion parameters between BL vs. ML, LS and MLS, and between LS v. MLS, we were unable to determine the histological basis for the changes in each individual parameter (e.g. associations with cell size, distribution, cytoplasmic ratio, or degree of necrosis in tumor tissues). Therefore, the correlation between each parameter and histological features is an area for future research. Third, the sample size for each tumor subtype is relatively small, and there is a diverse range of pathological types included, particularly in the benign lesion group. This potential bias in case selection may affect the final thresholds, limiting our ability to explore the correlation of various parameters with the Ki-67 index within smaller malignant subgroups. Future studies will require larger cohorts to clarify the diagnostic capabilities of the two non-Gaussian models in subgroups and their relationship with Ki-67. Fourth, additional non-Gaussian models, such as diffusion kurtosis imaging and intravoxel incoherent motion imaging, were not included for comparative analysis, which restricts our ability to explore the interrelationships among different diffusion parameters. Fifth, although this study utilized RESOLVE scanning as a replacement for single-shot echo-planar imaging for the acquisition of diffusion signals, the relatively longer acquisition time may introduce some motion artifacts. Therefore, future studies could use, for example, multi-slice simultaneous acquisition techniques to shorten the signal acquisition time, further improve the stability of the data, and increase the inter-observer agreement of quantitative parameters.

In conclusion, non-invasive, non-Gaussian FROC- and CTRW-based diffusion models are not only able to differentiate between malignant and benign tumors in the head and neck but can also provide additional information related to the heterogeneity of tumor tissues. Moreover, multiple diffusion parameters derived from these models were correlated with the level of Ki-67 positive expression in tumor histopathology. Therefore, the FROC and CTRW diffusion models and their derived parameters, especially α_CTRW_, are promising imaging tools and biomarkers for the differential diagnosis of lesions in the head and neck region.

## Data Availability

The raw data supporting the conclusions of this article will be made available by the authors, without undue reservation.
